# Management of tetralogy of Fallot in the pediatric intensive care unit

**DOI:** 10.3389/fped.2023.1104533

**Published:** 2023-06-08

**Authors:** Owen Hammett, Michael J. Griksaitis

**Affiliations:** ^1^ Paediatric Intensive Care Unit, University Hospital Southampton NHS Foundation Trust, Southampton, United Kingdom; ^2^ Dorset and Somerset Air Ambulance, South Western Ambulance Service NHS Foundation Trust, Exeter, United Kingdom; ^3^ Faculty of Medicine, University of Southampton, Southampton, United Kingdom

**Keywords:** tetralogy of Fallot, paediatric intensive care, management, congenital heart disease—cardiac, paediatric cardiology

## Abstract

Tetralogy of Fallot (ToF) is one of the most common congenital cyanotic heart lesions and can present to a variety of health care professionals, including teams working in pediatric intensive care. Pediatric intensive care teams may care for a child with ToF pre-operatively, peri-operatively, and post-operatively. Each stage of management presents its own unique challenges. In this paper we discuss the role of pediatric intensive care in each stage of management.

## 1. Introduction

Tetralogy of Fallot (ToF) is one of the most common cyanotic congenital cardiac lesions, with an incidence of 4 in every 10,000 live births (1). ToF is characterized by four anatomical features: ventricular septal defect (VSD); right ventricular outflow tract obstruction (RVOTO); an overriding aorta; and right ventricular (RV) hypertrophy (2). It is the degree of RVOTO that is of greatest interest to the pediatric intensivist.

Approximately 20% of patients with ToF also have pulmonary atresia (PA) (3), which is associated with further variation in pulmonary artery anatomy, including the absence of native pulmonary arteries and the presence of major aortopulmonary collateral arteries (MAPCAs) as the main source of pulmonary blood flow (4).

These variations in patients’ anatomy produce a continuum of physiological sequalae that pose ongoing challenges to clinicians in the management of this group pre- and post-operatively within a pediatric intensive care unit (PICU) setting.

Associated chromosomal anomalies for this patient group include 22q11 microdeletion and trisomy 21, 18, and 13 (5, 6), with the PICU team also needing to manage the associated features of these syndromes.

Before considering the specific physiology and management, it is vital to ensure that children with ToF presenting acutely to any healthcare provider are resuscitated using a standard “ABCDE” approach. However, an understanding of the underlying physiological factors that are unique to ToF may influence clinicians’ decision-making, for example, in ensuring optimization of physiology prior to induction of anesthesia.

Once immediate life-threatening emergencies have been managed, treatment of the specific physiological factors relating to ToF patients should be instigated. The different anatomical and physiological features for ToF are described below.

This article presents a discussion of the specific pre-operative and post-operative management of this patient group requiring intensive care.

## 2. Pre-operative management

Due to the four anatomic characteristics described above, mixing of oxygenated and deoxygenated blood between the pulmonary and systemic circulations occurs. Mixing most commonly occurs at the level of the VSD, typically with a right-to-left shunt causing systemic cyanosis (7). The degree of shunt is determined by the relative pressure gradient between the RV and left ventricle (LV), with RV pressure (and right-to-left shunt) increasing depending on the severity of RVOTO (8).

Pre-operatively, the majority of ToF patients are well and are managed in the community under pediatric and cardiology care until they have reached a suitable weight to undergo complete surgical repair. However, a small group of neonates and infants will present with profound hypoxia or significant “spelling,” or have a lesion that is dependent on a patent ductus arteriosus (PDA) to allow adequate pulmonary blood flow.

When dealing with these situations, the PICU team needs to consider the following factors:

- Duct dependent circulation
- Systemic vascular resistance (SVR)
- Pulmonary vascular resistance (PVR)
- Diastolic function
- Dynamic RVOTO from sub-valvular muscle bundles (“spelling”).

### 2.1. Duct dependent circulation

Neonates born with severe RVOTO, or pulmonary atresia, require a PDA to enable adequate pulmonary blood flow to allow oxygenation (8). Closure of the PDA leads to increased hypoxia and subsequent acidosis. The use of prostaglandin [alprostadil (prostin E1) or dinoprostone (prostin E2)] maintains the PDA in these cases of duct dependent circulation (9, 10). Side effects of the prostaglandin, namely apneas at higher doses, are likely to require PICU care.

Stenting of the PDA or the RVOTO may be an option through cardiac catheterisation to reduce the requirement for prostaglandin, providing more secure pulmonary blood flow while awaiting primary ToF repair (11). Although complete ToF repair is the aim, neonates who are duct dependent may require palliative intervention to enable them to grow to a suitable weight for corrective surgery. A modified Blalock–Thomas–Taussig (mBTT) shunt (connecting the subclavian artery to the ipsilateral pulmonary artery using a Teflon or Gore-Tex tube) allows for left-to-right blood flow, improving pulmonary blood flow (2, 12).

If an mBTT shunt is required, it is important to consider the potential complications when managing these patients post-operatively. The core principles of the post-operative course for mBTT shunts patients are to ensure adequate blood flow to both pulmonary and systemic circulations and to maintain shunt patency with anticoagulation.

Children with pulmonary overcirculation (high pulmonary blood flow) may present with relatively high oxygen saturation, edematous lungs on chest x-ray or ultrasound, low central venous saturation, and rising lactate. Pulmonary overcirculation can be managed by manipulating pulmonary and systemic vascular resistance in order to reduce blood flow to the lung (by increasing PVR) and ensure a balanced blood flow. It may be helpful to determine the Qp:Qs ratio to assist in directing appropriate therapies, with the aim of achieving a Qp:Qs ratio of 1:1–1.5 with systemic arterial oxygen saturation (SaO_2_) of between 75% and 80%.

Patients presenting with significant sustained desaturation and/or falls in end-tidal CO_2_ associated with a weaker or absent shunt murmur should be considered to have a blocked shunt (13). Blocked shunts following mBTT shunt palliation occurs in 4%–10% of patients immediately post-operatively (14). This is an emergency, as these patients are dependent on the shunt for pulmonary blood flow. This scenario requires increasing SVR, decreasing PVR, and increasing cardiac output to promote pulmonary blood flow, in addition to ensuring patients are adequately anti-coagulated.

### 2.2. Systemic vascular resistance (SVR)

Increases or decreases in SVR may have a profound effect on ToF physiology. In the presence of RVOTO, a reduction in SVR (vasodilation) will increase right-to-left shunt by decreasing LV end-diastolic pressure (LVEDP), and therefore increase the pressure gradient between the RV and LV cavities (7). This can be seen in situations including, but not limited to, vasodilated sepsis. In contrast, an increase in SVR (vasoconstriction) will achieve the opposite by increasing LVEDP, thereby reducing right-to-left shunt. Increasing SVR forms the basis for the management of spelling episodes, which is discussed specifically later in this article.

Vasoconstriction (and subsequent increase in SVR) can therefore be used to reduce right-to-left shunt in extreme physiological situations (15). α agonists (for example phenylephrine or noradrenaline) or other vasopressor agents (such as vasopressin) can be used to increase SVR in patients presenting with profound hypoxia or who have undergone a reduction in SVR, for example, during or following induction of anesthesia or with other pathologies such as sepsis (7). The use of cooling to manage temperature offers an additional method to increase SVR by inducing peripheral vasoconstriction.

### 2.3. Pulmonary vascular resistance (PVR)

PVR is usually not a significant issue in pre-operative cases of ToF due to pulmonary circulation being protected by the RVOTO. It is more relevant in a “pink” Fallot's, with little RVOTO. However, high PVR increases the degree of right-to-left shunting by increasing the RV afterload, in addition to the RVOTO, to further reduce pulmonary blood flow. This is probably of secondary importance to SVR when dealing with profound cyanosis in ToF, but reducing PVR can help with significant spelling episodes.

PVR can be reduced in order to promote pulmonary blood flow in a case of cyanotic uncorrected ToF, in parallel to increasing SVR. Management of PVR needs to be conducted in conjunction with SVR management, because (as previously mentioned) PVR as an isolated problem in a pre-operative ToF patient is rare due to protection of the pulmonary vasculature from high pulmonary blood flow by the RVOTO. However, marginal gains during significant spells can make a difference to the patient's oxygen saturation.

PVR reduction can be achieved via:

• Administration of a high fraction of inspired oxygen (FiO_2_) (acts as a pulmonary vasodilator, and improves oxygen transfer across the alveolar basement membrane in the presence of any associated VQ mismatch);
• Intubation and ventilation to control PaCO_2_ to normocarbia (high CO_2_ is a pulmonary vasoconstrictor) and ventilation strategies to recruit to the patient's functional residual capacity (FRC) volume to minimise PVR;
• Administration of pulmonary vasodilators such as inhaled nitric oxide (iNO);
• Sedation and muscle relaxation to reduce oxygen consumption (7, 13).

### 2.4. Diastolic function

RV diastolic function is often impaired in ToF patients due to RVH causing poor lusitropy (rate of myocardial relaxation), leading to a reduction in baseline diastolic function (7).

Children with ToF are dependent on an increased venous return due to the increased RV end-diastolic pressure resulting from RVH. The demand for adequate venous return exists prior to surgery and persists following repair for several days to weeks; however, this does also impact the pre-repair patient. Optimising pre-load by ensuring appropriate volume resuscitation is vital in assuring ventricular filling, pulmonary blood flow, and cardiac output.

### 2.5. Dynamic RVOTO with sub-valvular muscle bundles (“spelling”)

In ToF, the RVOTO is often anatomically fixed at the valve level, but it can be worsened by sub-pulmonary valve muscle bundles that have the potential to contract or spasm, which can cause worsening acute arterial desaturation (14). Hyper-cyanotic episodes, known as “spells,” are episodes of acute deterioration and desaturation are classically associated with an uncorrected ToF with a closed PDA. Spelling episodes occur due to infundibular muscle spasm and/or alternation in the SVR:PVR ratio causing a sudden reduction in pulmonary blood flow and increasing right-to-left shunt (2, 10).

**Figure 1:**
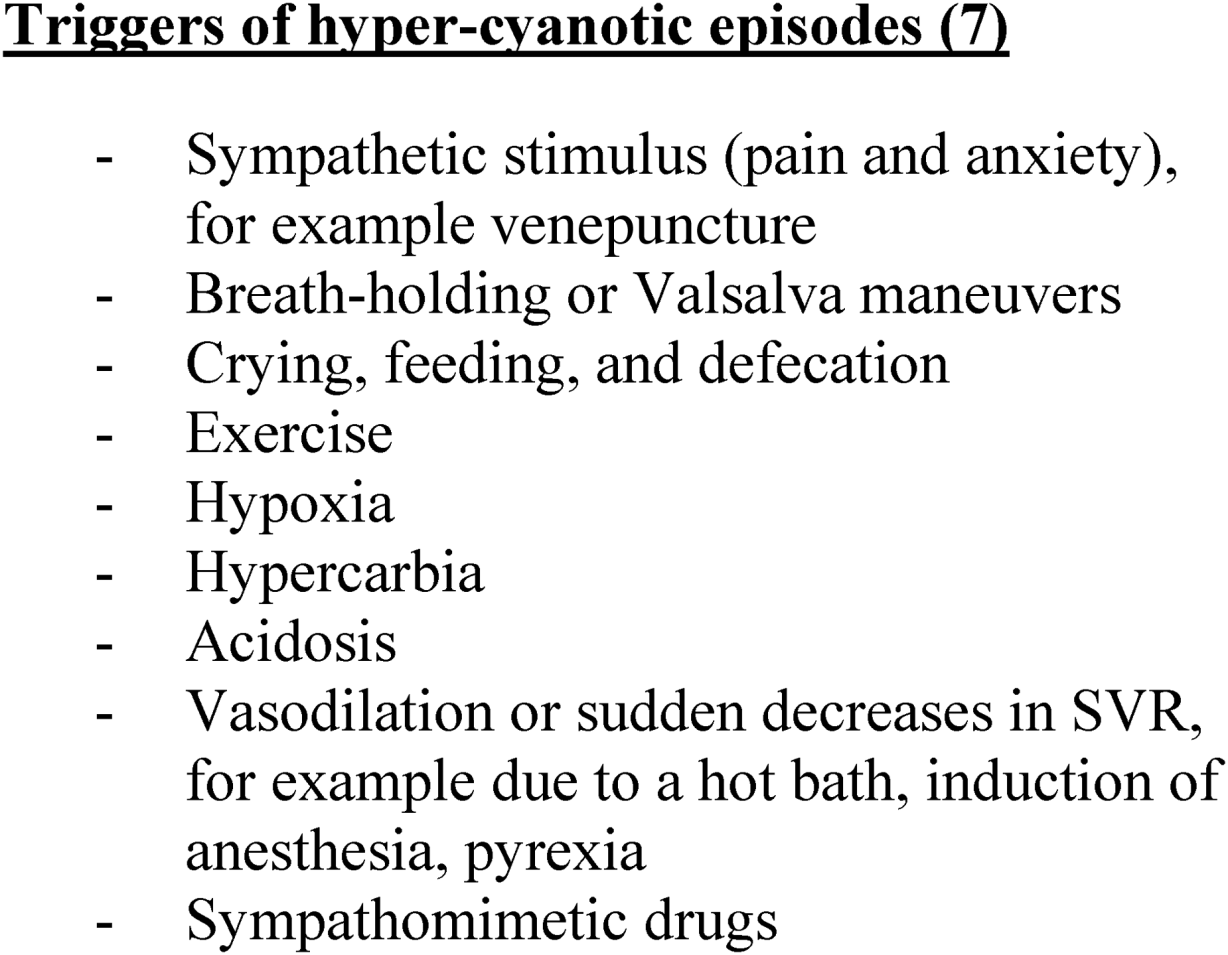
Summary of triggers that can promote hypercyanotic episode..

**Figure 2:**
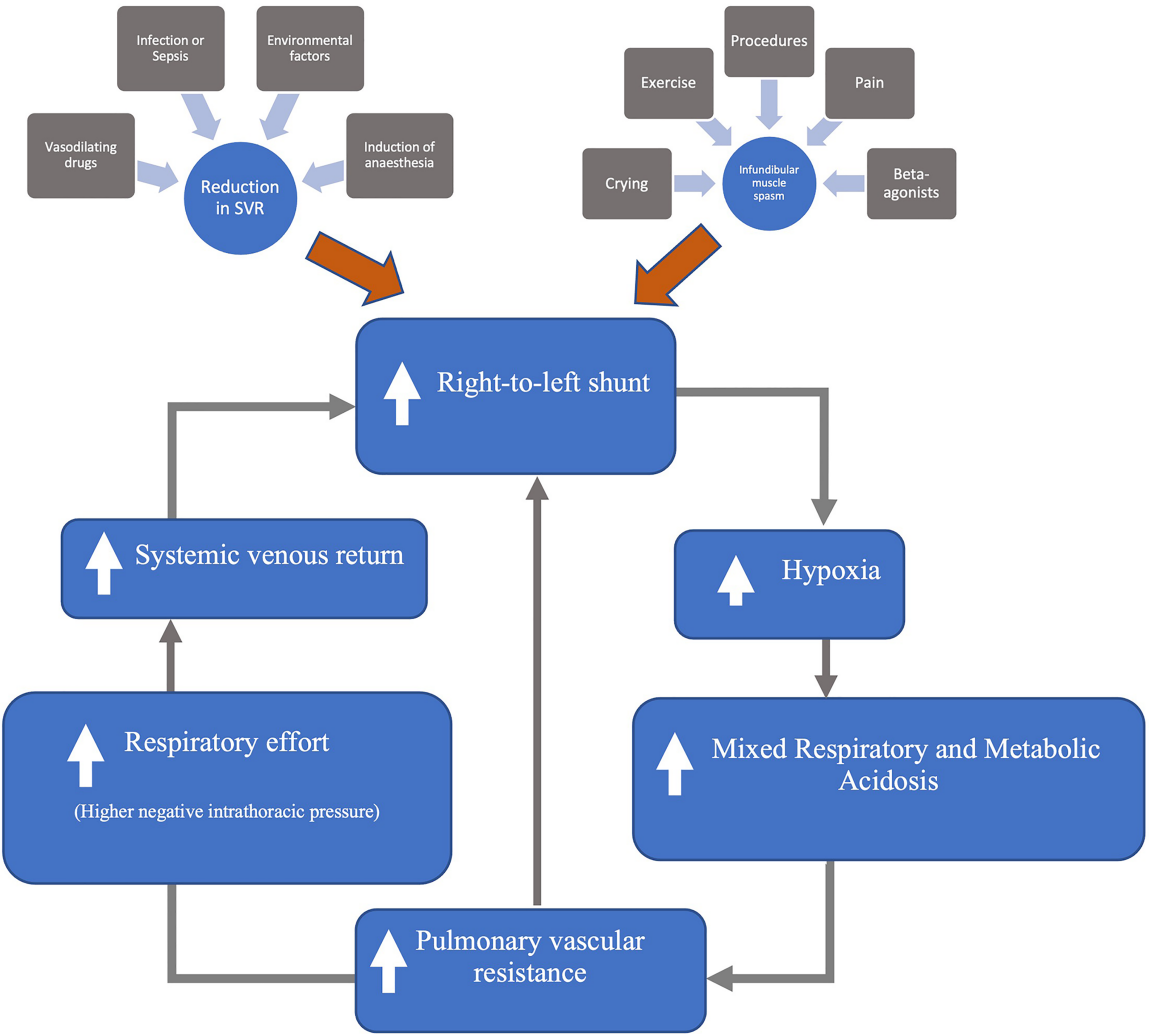
Mechanism of hyper-cyanotic spells [modified from Wilson et al. (7)].

Sympathetic stimulation is often the precipitating factor; however, other factors can trigger these episodes (see Figure 1). The associated cyanosis and acidosis that ensues can cause a vicious cycle of further shunting and subsequent deterioration (see Figure 2). Where possible, it is vital to avoid these triggers. Furthermore, any illnesses leading to hypovolemia (for example, diarrhea, vomiting, or excessive use of diuretics) can precipitate spelling. Children presenting with spelling should be appropriately volume-resuscitated if signs of hypovolemic shock are present.

Spelling children will present cyanosed and tachycardic and with cerebral agitation. Reduction in consciousness level or seizures can occur in patients with severe spelling attacks (16). Cerebral vascular accidents are a potential risk in this patient group. Spelling episodes are often self-limiting, but supportive management may be necessary in the PICU if the spell does not terminate. Propranolol is often used in children at risk of spelling.

The goals in managing severe acute hyper-cyanotic episodes are (1) supportive treatment for the patient’s airway, breathing, circulation, and disability (reducing agitation); and (2) reduction of right-to-left shunt by breaking the spelling cycle by reducing dynamic RVOTO, decreasing PVR, and increasing SVR to raise LVEDP (7).

The management of a spell includes:

• Administration of high-flow oxygen (needed to reduce the severity of the hypoxia);
• Ensuring adequate analgesia (e.g., morphine);
• Bringing the knees to the chest (aiming to improve venous return);
• Fluid bolus (improving venous return);
• Increasing SVR (e.g., phenylepherine bolus or infusions of noradrenaline/vasopressin);
• Intubation & ventilation (to sedate, reduce muscle spasm, and reduce oxygen consumption)
  ○ Caution is needed, as induction of anesthesia can lower SVR, which may worsen cyanosis
• Use of β-blockers to reduce RVOT muscle bundle spasm;
• In cases of refractory hypoxia despite the above management, it is necessary to consider:
  ○ Extracorporeal life support (ECLS)
  ○ Surgical intervention (e.g., BT shunt or full repair).

## 3. Post-operative management after full ToF repair

The aim for ToF patients is full surgical correction with VSD closure and relief of RVOTO, leaving an anatomically normalized heart. The age of repair varies, ranging from primary neonatal repair to young infants. There is debate regarding the most appropriate surgical management; however, there are some general concepts that are relevant to the paediatric intensivist (17).

ToF repair requires cardiopulmonary bypass (CPB), and the general effects of CPB, cross-clamp time, bleeding, low cardiac output syndrome, and systemic inflammatory response should be managed following return to the PICU. Whilst rare in a ToF repair, significant low cardiac output (due to any cause) may require extracorporeal life support (ECLS) to support oxygen delivery. In these cases, early consideration should be given to cardiac catheterization or cardiac CT.

A typical fully corrected ToF repair patient usually experiences non-complex post-operative recovery and is often extubated early within 12–24 h, often requiring a minimal amount of intervention in the form of some volume requirement (usually <20 ml/kg in the first post-operative night) and the use of cardiovascular support, as discussed below. Pleural drains are often left *in situ* for an additional 24–48 h post-operatively due to the risk of pleural effusions. If complications occur, then the post-operative clinical course and PICU stay is prolonged.

There are some specific complications to ToF repair that can occur; these include:

- Restrictive RV physiology;
- Arrhythmias;
- Residual lesions.

### 3.1. Restrictive RV physiology

Restrictive RV physiology is defined as antegrade end-diastolic forward flow into the pulmonary artery (18). This antegrade flow is caused by decreased RV compliance. There is evidence for several factors increasing the likelihood of restrictive RV physiology post-operatively, including baseline pre-operative oxygenation saturation, transannular patch repair, long bypass and cross-clamp time, and ventricular hypertrophy >5 mm (19).

The management of restrictive RV physiology post-ToF repair on the PICU is a common and important issue, as those with this physiology can have a low cardiac output and experience prolonged PICU admissions.

The complications of restrictive RV physiology include:

• Low cardiac output state;
• RV dysfunction (both systolic and diastolic);
• High central venous pressures;
• Pleural effusions (can also develop chylothoracies);
• Ascites;
• Liver and renal impairment.

The management of this specific physiological state involves maintaining adequate RV pre-load and promoting diastolic filling in the RV (20). This can be achieved with the use of volume resuscitation or by improving venous return using noradrenaline or vasopressin (21). The use of milrinone to enhance RV function and support ventricular lusitropy in order to improve diastolic relaxation is common (22). Furthermore, it is important to increase SVR using α agonists (e.g., noradrenaline) to enable an increase in systemic perfusion without significantly increasing RV afterload and to maintain systolic pressure and coronary and reno-vascular perfusion pressures. This can also help to overcome the vasodilatory effect and afterload reduction of the milrinone. Avoiding tachycardia will also improve RV filling. This can be achieved by trying to avoid the use of inotropes, through use of vasopressors instead.

Many children with restrictive RV physiology have a positive fluid balance due to the need for fluid administration to maintain RV pre-load. They can also develop pleural effusions and ascites due to the high central venous pressures and fluid administration with a low urine output. This additionally leads to general tissue edema and can also worsen respiratory function. This group of children may require renal replacement therapies (RRTs), such as peritoneal dialysis (23).

The theory of cardiopulmonary interactions would suggest that early extubation is beneficial for RV function, and this physiological theory would fit with a restrictive RV. However, the benefits of positive pressure ventilation (PPV) should be considered within the full clinical picture, and often a very sick child post-ToF repair benefits from PPV in terms of reduced oxygen consumption, allowing cooling, RRT administration, and improvement of oxygen delivery. Finding the balance can be difficult; however, multi-professional discussion with cardiology and cardiac surgical colleagues can support the decision-making process.

### 3.2. Arrhythmias

Arrhythmias are common following ToF repair, with junctional ectopic tachycardia (JET) being the most common post-ToF repair. Sinus rhythm with atrio-ventricular synchrony should be maintained to provide adequate cardiac output.

**Figure 3:**
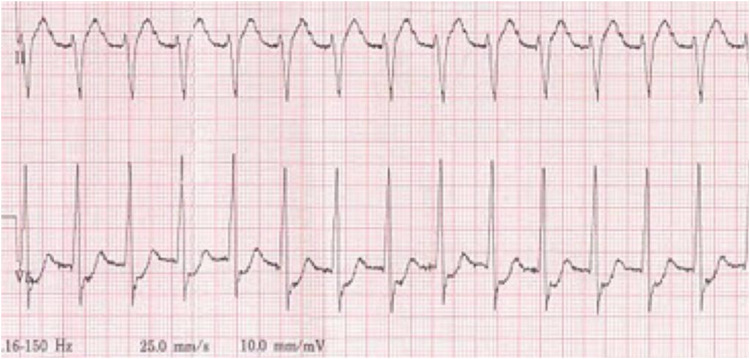
Junctional ectopic tachycardia.

JET can be recognized by atrial and ventricular (AV) dissociation with an acceleration in ventricular rate, hemodynamic compromise, signs of low cardiac output, and development of cannon waves on the central venous pressure waveform. A 12-lead ECG (+/− an atrial ECG) will confirm the diagnosis by showing a narrow complex tachycardia with AV dissociation and a faster ventricular rate (24). The rate of the ventricular component of JET can determine the clinical significance for cardiac output. Patients suffering from JET with an underlying bundle branch block may mimic ventricular tachycardia (VT) (24). Other tachyarrhythmias, such as SVT, can also occur. An example of JET is shown in Figure 3.

The management of JET, and many other tachyarrythmias, involves reducing sympathetic drive by ensuring adequate sedation, reducing inotrope/chronotrope use, and ensuring electrolytes (namely, potassium, calcium, and magnesium) are maintained within normal limits. Cooling to normothermia can be helpful. Obtaining rate control of the JET using clonidine or dexmetatomadine and/or amiodarone may subsequently allow overdrive atrial pacing to allow AV synchrony to be achieved, if epicardial pacing wires have been inserted in theater. The amount of intervention required depends on the clinical impact on the patient's oxygen delivery and cardiac output. Some children can tolerate JET at a lower rate and others may need ECLS. This decision is made on a case-by-case basis using parameters such as blood pressure, lactate, urine output, and central venous oxygen saturations. The natural longevity of JET is that it will resolve in 10–14 days. It is important to note that JET cannot be cardioverted.

Complete heart block can also occur in 3%–5% of patients, requiring dual-chamber epicardial pacing with recovery expected within 10 days. If this is not the case, permanent pacemaker insertion should be considered. It is important for the intensive care team to monitor pacing thresholds in these children, as rising thresholds may mandate earlier insertion of PPM or replacement of epicardial wires.

### 3.3. Residual lesions

In all congenital cardiac surgery, some residual lesions can occur, and in ToF this can include:

• Residual VSD (often small and insignificant, but keep an open mind if signs of high Qp:Qs are present);
• Residual RVOTO;
• Pulmonary valve regurgitation (expected after a transanular patch repair).

Residual RVOT stenosis will limit pulmonary blood flow. In a case of repaired ToF in which the patient now has a closed VSD, there is no ability for right-to-left shunting. If residual RVOTO is demonstrated, an early discussion with cardiology and the cardiac surgical team is required to determine the best treatment strategy moving forward.

In some ToF patients, when the pulmonary valve is incompetent or the RVOTO has been resected and repaired using a transannular patch, leaving a residual or absent pulmonary valve, blood flow during diastole can regurgitate back into the RV (25). This free-flowing pulmonary valve regurgitation causes volume overload onto the RV, which is associated with RV dilation, biventricular dysfunction, and arrhythmias. These are often well tolerated in early life, but this does impact pulmonary blood flow and ventricular filling.

Early consideration should be given to cardiac catheterization or cardiac CT to look for residual lesions if the clinical course is not progressing as expected.

## 4. Long-term issues and management considerations

It is uncommon for pediatric intensivists to be involved with corrected ToF patients following discharge from the PICU or hospital. Nevertheless, due to ongoing complications, they may be involved in the acute management of children presenting to the ED or pediatric cardiology wards. These complications can include requirement for pulmonary valve replacement to reduce pulmonary regurgitation, pleural effusions, heart failure, arrhythmias, and cardiac arrest (26).

Corrected ToF patients are at high risk of ventricular arrhythmias, including VT and supraventricular tachycardia (SVT). Due to this risk, they may present to EDs in cardiac arrest with a VT etiology; management should involve ensuring that Resuscitation Council guidelines have been followed and that electrolyte imbalances have been corrected.

## 5. Conclusion

ToF is one of the most common forms of congenital cardiac cyanotic lesion, and patients with this condition are admitted to PICUs following post-operative repair; however, those in extremis may present pre-operatively. It is vital for all pediatric critical care clinicians to understand the anatomical and physiological differences in ToF to enable them to apply appropriate interventions and to offer support to the referring hospitals when providing remote advice prior to retrieval.

Post-operatively, there are several complications that are common across all cardiac patients who have received surgical intervention. Furthermore, there are specific complications relevant to ToF patients that should be considered when managing immediate and ongoing post-operative care within the PICU. It is also important to note that corrected ToF patients may present acutely to hospital as children and young adults, and PICU clinicians may be required to support the care delivered to these patients.
